# Pilocytic Astrocytoma: An institutional perspective over the last decade

**DOI:** 10.12669/pjms.41.13(PINS-NNOS).13394

**Published:** 2025-12

**Authors:** Shah Khalid, Noman Ahmed, Fatima Zahra, Uzair Ahmed, Ather Enam

**Affiliations:** 1 Shah Khalid, MBBS, FCPS.Department of Neurosurgery, Aga Khan University Hospital (AKUH), Karachi, Pakistan; 2 Noman Ahmed, MBBS.Department of Neurosurgery, Aga Khan University Hospital (AKUH), Karachi, Pakistan; 3 Fatima Zahra,Medical Student, Medical College, Aga Khan University Hospital (AKUH), Karachi, Pakistan; 4 Uzair Ahmed Medical Student, Medical college, Aga Khan University Hospital (AKUH), Karachi, Pakistan; 5 Ather Enam, MD, PhD, FRCSI, FRCSC, FRCSG FACS. Diplomate American Board of Neurosurgery,Scientific Director, Juma Research Laboratory, Aga Khan University Hospital (AKUH), Karachi, Pakistan

**Keywords:** Pilocytic Astrocytoma, Clinical & radiologic features, Survival, Prognostic factors

## Abstract

**Background and Objective:**

Pilocytic astrocytoma primarily affects children, commonly originates in the cerebellum, and generally has a favorable prognosis. The prognostic significance of patient age, tumor location, and extent of resection remains debated. This retrospective study aimed to evaluate clinicopathological and radiological features, identify prognostic factors, and assess progression-free survival at 1 and 5 years and overall survival at one, five and 10 years.

**Methodology:**

This retrospective study was conducted at The Aga Khan University Hospital (2013–2022) on patients with histologically confirmed pilocytic astrocytoma. Patients with less than one year of follow-up were excluded. Data on demographics, clinical presentation, tumor characteristics, extent of resection, adjuvant therapy, and outcomes were collected. Survival outcomes were analyzed using Kaplan-Meier and Cox regression methods, with statistical significance set at p < 0.05.

**Results:**

In the study of 101 patients, 53 (52.5%) were male and 48 (47.5%) were female. Of these, 59 were pediatric patients (<14 years), 35 were aged 15-39 years, and 7 were over 39, with a mean age of 16.9 years. The median pre-operative Lansky/KPS score was 80. The cerebellum was the most common site for tumors, and most tumors were solid-cystic (69.6%), while 14.5% were purely cystic. The mean preoperative tumor volume was 52.9 ml (SD 62.5). Hydrocephalus was present in 49.5% of patients, and leptomeningeal dissemination was seen in five patients. Gross total resection (GTR) was achieved in 37 (43.0%), near-total resection (NTR) in 7 (8.1%), subtotal resection (STR) in 38 (44.1%), and four patients (4.6%) had biopsy only. As an adjuvant treatment, 8 patients received both chemoradiation, four patients had received only chemotherapy, and 4 had received radiation only. Two patients died within 30 days after surgery, and 22 had recurrence/progression. The 1-year survival rate was 92%, 5-year survival was 87%, and 10-year survival was 83%. The 1-year progression-free survival (PFS) was 50%, and 5-year PFS was 10%. Kaplan-Meier analysis showed trends for better overall survival (OS) with GTR or NTR, cystic tumors with nodules, pediatric patients, absence of leptomeningeal dissemination, and microvascular proliferation, but these differences were not statistically significant

**Conclusion:**

This study of pilocytic astrocytoma over the past decade in a low-and-middle-income country highlights survival rates (1-year, 5-year, 10-year) reported for the first time in this population. Survival trends varied by age, leptomeningeal disease, extent of resection, and radiological tumor patterns. Cox regression identified radiological patterns, CNS infections, and endocrinological dysfunction as key predictors of overall survival.

## INTRODUCTION

Pilocytic astrocytoma, a prevalent glial tumor, primarily affects children aged 5 to 14 years. Its incidence is notably higher in this age group, constituting approximately 11.4% of cases among individuals aged 0 to 19, in contrast to a mere 0.8% among young adults.^1^ According to the WHO classification of brain tumors, pilocytic astrocytoma is categorized as a Grade-I tumor and can manifest anywhere along the neuroaxis.^2^ However, it most commonly originates in the cerebellum, accounting for around 30% of all posterior fossa tumors.^3^ Additionally, these tumors are found in various other locations such as optic pathways, brainstem, cerebral hemispheres, hypothalamus, and the third ventricle.^4^ Despite their diverse locations, the prognosis for pilocytic astrocytoma is generally favorable due to its slow-growing nature. Pediatric patients exhibit an overall survival rate exceeding 90% after complete surgical resection of the tumor.^1^ However, complete resection may not always be feasible due to the tumor’s variable location, leading to residual lesions that can impact prognosis through surgical complications and tumor recurrence.^5^ Despite favorable prognosis, different factors including pathological features, anatomic location, and extent of tumor resection have been reported to influence the prognosis of PA after resection.^3^ A study by Stuer C et al revealed unfavorable outcomes among adult patients diagnosed with Pilocytic Astrocytoma.^6^ However, some evidence indicates that adults with PA may experience the favorable prognosis typically observed in children.^7^-^9^ Furthermore, patients diagnosed with the pilomyxoid variant and those experiencing tumor invasion into surrounding structures generally exhibit poor prognosis.^10^ The prognostic significance of patients age, tumor location as well as extent of tumor resection has been a subject of debate, drawing from previously published small series and population-based studies.^6^,^11^-^13^ Pilocytic Astrocytoma, though commonly encountered, very little and age limited information is available in the literature about tumor characteristics and outcome in our population.^14^ This retrospective study aims to identify pilocytic astrocytoma’s clinical and histopathological features, focusing on its characteristic radiological findings and prognostic factors. Additionally, it seeks to evaluate the surgical outcomes, including progression-free survival rates at one and five years and overall survival rates at one, five and 10 years. These findings will assist clinicians in better understanding the disease process, determining optimal management strategies, predicting outcomes, identifying various prognostic factors, and establishing the appropriate duration and frequency of follow-up. This can help reduce the psychological and financial strain on patients and their families by providing clarity on expected outcomes and avoiding unnecessary follow-up visits.

## METHODOLOGY

This study was a retrospective cohort study conducted at the Aga Khan University Hospital, a tertiary, referral center within the region from January 2013 to December 2022 . A non-probability convenience sampling technique was used to recruit patients. Data were recorded including age at diagnosis, gender, presenting signs and symptoms, pre-operative KPS/Lansky score, tumor location, radiologic features of tumors with other associated findings (invasion of nearby structures, absence or presence of hydrocephalus, leptomeningeal dissemination, and mass effect), extent of resection, history of CSF diversion, adjuvant therapy (RXT, CTX) post-operative complications and post-operative KPS/Lansky score on last follow-up visit. Tumor location and radiologic features were confirmed by reviewing pre-operative imaging studies. Histopathological characteristics were recorded from EMR. The extent of surgical resection (Biopsy, STR, NTR, or GTR) was determined by reviewing the surgeon’s intra-operative notes and report of early post-operative imaging studies (MRI). The record of each follow-up visit especially the patient’s clinical condition, occurrence of new or progression of existing signs and symptoms, imaging findings with a special focus on recurrence or progression of residual disease, and history of revision surgery were documented. after ***Ethical Approval:*** Ethical Review Committee approval No. (2024-10363-30113), Dated: July 26, 2024.

### Inclusion criteria

- Patients of either gender or any age, with histologically confirmed pilocytic astrocytoma (PA).
- Patients with a mean follow-up of ≥1 year.

### Exclusion criteria

- Patients with a minimum follow-up duration of < 1 year.
- Patients with incomplete records.

### Data analysis technique:

SPSS version 23 used for collection and analysis. All continuous variables are reported as mean/median depending upon the normality of data. Clinical and pathological features are reported as frequencies. Survival analysis including overall survival (OS) and progression-free survival (PFS) is done using the Kaplan-Meier method and survival differences between groups are analyzed by the log-rank test. Results are considered significant at a p-value of < 0.05. Cases with missing data are excluded from respective analyses. Cox regression analysis was conducted for predictors of survival within the cohort, with 95% Cis reported and hazard ratios.

## RESULTS

Within the last 10 years, 101 patients were included in the study, among whom 59 were pediatric cases, 35 were adolescents and young adults, and 7 belonged to the adult population. The pre-operative median Lansky/KPS score of the cohort was 80 with a mean age of 16.9 years (S.D. 12.7). Five patients had a previous history of comorbid disease – 2 with neurofibromatosis, one with a previously diagnosed and treated pituitary adenoma, 1 with scoliosis, and 1 with thalassemia minor. As shown in Table-I, most patients were male (52.5%) with tumors most likely within the cerebellar (43.6%) and lobar (37.6%) regions. Radiological patterns of PA were evaluated with most presenting as solid-cystic (69.6%) and purely cystic (14.5%) tumors. Pre-operative hydrocephalus was present within 49.5% of patients and leptomeningeal dissemination in 5 patients. The mean preoperative tumor volume was 52.9 ml (S.D. 62.5). 37.6% of the patients required intraoperative CSF diversion. The predominant histological characteristic was the presence of Rosenthal fibers and eosinophilic granular bodies. Adjuvant chemotherapy alone (vinblastine) was administered to 4 pediatric cases, adjuvant radiotherapy alone to 4 patients, and both chemo-radiation to 8 patients.

**Table-I:** Patient demographics, symptoms and signs, tumor location, radiological patterns, histopathological features, adjuvant treatment and CSF diversion within cohort.

Gender	Female	48	47.5%
	Male	53	52.5%
Age	Pediatric	59	58.4%
	Adolescent & Young adult	35	34.6%
	Adult	7	6.9%
Signs & Symptoms at the time of presentation	Raised ICP	63	62.3%
	Cerebellar dysfunction	38	37.6%
	Seizures	22	21.8%
	Motor Deficit	19	18.8%
	Cranial Nerve Palsy	7	6.9%
	Sensory Deficit	1	0.9%
Tumor location	Infratentorial	51	50.5%
	Cerebellum	44	43.6%
	Brainstem	5	5.0%
	Spinal cord	2	2.0%
	Supratentorial	50	49.5%
	Lobar	38	37.6%
	Basal ganglia/Thalamus	7	6.9%
	Intraventricular	4	4.0%
	Pineal region	1	1.0%
Radiological pattern	Solid-cystic	48	69.6%
	Cystic	10	14.5%
	Solid	8	11.6%
	Cyst with nodule	3	4.3%
Preoperative Hydrocephalus		50	49.5%
Leptomeningeal dissemination		5	4.9%
Predominant Histologic Features	Mucous/fibrillary background	29	28.7%
	Necrosis	3	3.0%
	Microvascular proliferation	14	13.9%
	Eosinophilic granular bodies	63	62.4%
	Calcifications	10	9.9%
	Microcysts	24	23.8%
	Rosenthal Fibers	77	76.2%
Adjuvant treatment	Chemotherapy only	4	3.9%
	Radiation only	4	3.9%
	Both	8	7.9%
CSF Diversion for Pre-resection HCP	Before tumor resection (as a separate procedure)	12	11.9%
	VP Shunt	12	11.9%
	EVD	0	0.00%
	At the time of tumor resection	38	37.6%
	EVD	34	89.5%
	VPS	4	10.5%
CSF Diversion for Post-resection HCP	After Tumor resection (as a separate procedure)	14	13.8%
	EVD	1	7.1%
	VPS	13	92.8%

Table-II depicts the operative outcomes of Pilocytic astrocytoma surgery. In majority of cases there was a gross total (GTR) or sub-total (STR) resection, 43% and 44.1% respectively. On molecular analysis of select cases, BRAF V600E immunostaining was negative in 3 cases, and KIAA1549Ex16 BRAFEx9 fusion was positive in 2 cases. In outcomes, early death (within 30 days of operation) was recorded in 2 patients, and progression/recurrence in 22 patients. On survival analysis, median OS was highest in AYA (Adolescents and Young Adults) at 5.79 years with PFS of 2.08 years. The 1-year-survival rate for the overall cohort was 92%, 5-year-survival 87%, and 10-year-survival of 83% (Table-III).

**Table-II:** Intraoperative and postoperative outcomes

Extent of resection	Partial Resection/Biopsy	4	4.6%
	GTR	37	43.0%
	NTR	7	8.1%
	STR	38	44.1%
Post-Operative Complications	infarct/hemorrhage	6	5.9%
	Postoperative Endocrine dysfunction (DI/Electrolytes dysfunction/DKA)	7	6.9%
	Postoperative CNS infection	5	5.0%
	Postoperative wound complications (infection, pseudo meningocele, dehiscence)	2	2.0%
	Postoperative Neurological deficit/Seizures	23	22.8%
	Postoperative systemic infection (Sepsis/UTI/GI/Respiratory)	5	5.0%
In-hospital mortality		2	1.9%
Progression/Recurrence		22	21.8%
Re-resection		12	11.8%

**Table-III:** Overall and Progression-free Survival of the cohort

		Overall Survival (in years)	Progression-free Survival (in years)
		Median	Median
Age	Pediatrics	5.02	1.55
	Adolescents and Young Adults (AYA)	5.79	2.08
	Adults	4.41	2.43
Tumor Location	Infratentorial	5.42	2.98
	Brainstem	4.39	1.32
	Cerebellum	5.54	5.13
	Spinal	8.06	.
	Supratentorial	5.30	.93
	Lobar	5.79	.93
	Ventricular	3.89	
	Basal ganglia/Thalamus	3.19	
	Pineal	.99	.77
Extent of resection	Biopsy	4.10	.
	GTR	3.76	1.55
	NTR	8.02	2.43
	STR	5.71	2.27

**Fig.1:**
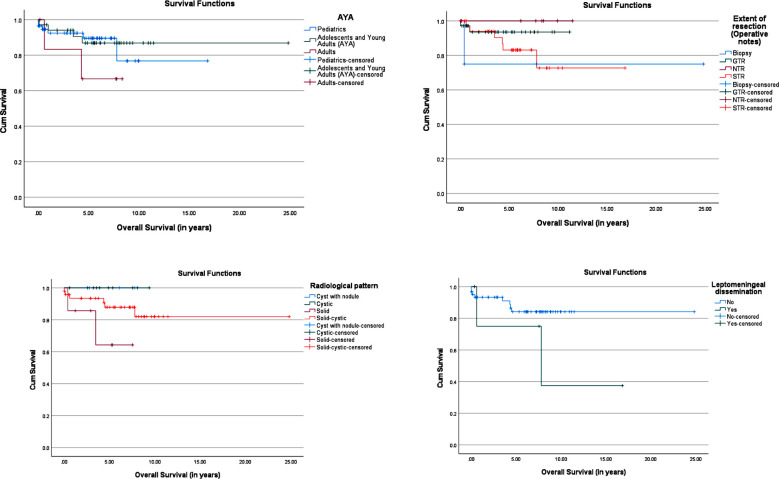
Kaplan-Meier curves showing overall survival predicted by variable factors.

**Fig.2:**
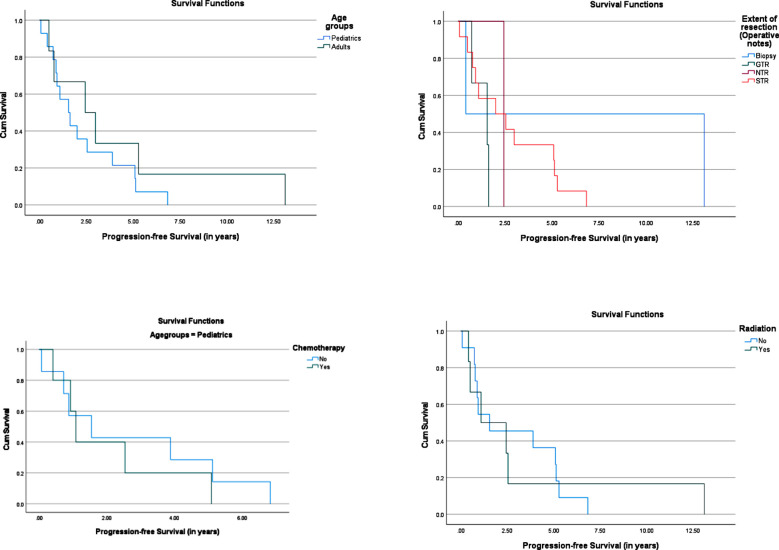
Kaplan-Meier curves showing factors predicting progression fee survival (PFS).

Fig.1 shows Kaplan-Meier curves, that suggest improved OS trended for GTR or NTR, cystic/cyst with nodule pattern, no leptomeningeal dissemination, microvascular proliferation, and pediatric patients, although these did not show statistically significant differences. 1-year-PFS was 50% and 5-year-PFS for PAs was 10% for the cohort. On evaluation of risk factors according to Kaplan-Meier curves (Fig.2), there were no significant differences or trends noted. Cox regression analysis (Table-IV) showed adults, GTR, solid-cystic, postop CNS infection, postop endocrine dysfunction, postop infarct/hemorrhage, preop KPS/Lansky, preop visual symptoms, leptomeningeal disease, cerebellar, pineal, ventricular, and supratentorial location to be significant predictors for OS on univariate analysis.

**Table-IV:** Univariate Cox regression analysis evaluating predictors for overall survival

		Hazard Ratio	p-value	95% CI
Age Groups				
	Pediatrics	(reference)		
	AYA	0.88	0.84	0.24 – 3.13
	Adults	2.67	0.23	0.54 – 13.25
EOR				
	Partial Resection/Biopsy	(reference)		
	GTR	0.22	0.22	0.02 – 2.48
	NTR	6.55e-17	1	
	STR	0.52	0.54	0.06 – 4.31
Radiological pattern				
	Cyst with nodule	(reference)		
	Cystic	8.84e-07	1	.
	Solid	7.20e+09	.	.
	Solid-cystic	2.28e+09	<0.001	4.39e+08 - 1.19e+10
Postop CNS infection		8.07	0.002	2.18 – 29.95
Postop Endocrine dysfunction		10.04	<0.001	2.99 – 33.61
Postop infarct/hemorrhage		3.04	0.15	0.66 – 13.96
Preop KPS/Lansky		0.94	0.14	0.86 – 1.02
Preop visual symptoms		3.72	0.025	1.18 – 11.74
Leptomeningeal disease		3.05	0.16	0.65 – 14.38
Location				
	Basal ganglia/Thalamus	(reference)		
	Brainstem	0.99	0.99	0.06 – 15.97
	Cerebellum	0.19	0.18	0.02 – 2.12
	Lobar	0.48	0.52	0.05 – 4.38
	Pineal	8.22	0.14	0.49 – 138.61
	Spinal	1.48e-16	1	.
	Ventricular	4.8	0.18	0.48 – 47.5
	Infratentorial	(reference)		
	Supratentorial	3.52	0.06	0.94 – 13.18

## DISCUSSION

Pilocytic astrocytoma is a glial neoplasm most often seen in pediatric patients, mainly located in the infratentorial compartment. Our study noted this, as the mean age of our cohort was 16.9 years, and approximately 50.5% of the tumors were in the infratentorial compartment. Our study reported a higher mean age when compared to the previous studies by Krieger et al and Malik et al.^15^,^16^

Supratentorial pilocytic astrocytomas are relatively less common compared to their infratentorial counterparts. In our study, we reported 49.5% of cases, mostly in the cerebral cortex while 50.5 percent of cases were found in the infratentorial compartment. Less commonly, these tumors have been found in the region of basal ganglia, thalamus, pineal gland, and inside the ventricles. Our cohort found 4 percent of the tumors inside the ventricular system. In a case series, Zuccaro and Sosa et al reported 54 intraventricular tumors, which included choroid plexus papillomas, ependymomas, astrocytoma and subependymal giant cell astrocytoma.^17^

As reported in the literature, the most common presentations include features of raised intracranial pressure and seizures.^18^,^19^ In our cohort, the most frequent clinical signs and symptoms were related to raised intracranial pressure (nausea, vomiting, visual disturbances), accounting for approximately 62.3% of cases. This was followed by signs and symptoms of cerebellar dysfunction, observed in approximately 37.6% of patients. The lower incidence of seizures compared to previous literature in our cohort is likely attributable to the relatively lower proportion of supratentorial PA and the slightly higher proportion of infratentorial cases.

The radiological appearance of PA is variable. According to Murray et al, 4 predominant patterns have been described; 1. Mass with a non-enhancing cyst and enhancing mural nodule; 2. Mass with an enhancing cyst wall and enhancing mural nodule; 3. Necrotic mass with central non-enhancing zone; 4. Solid mass without a cystic component.^20^,^21^ We reported 69.6% of solid-cystic patterns in our group of patients. This pattern corresponded to an increased overall survival rate; however, the results were statistically insignificant.

The presence of leptomeningeal disease has been identified as one of the negative predictors of overall survival in previously reported studies. According to Mazloom et al, 58 patients from 26 different studies had leptomeningeal disease either at presentation or diagnosed later during the treatment. The median survival time was 65 months for individuals with pilocytic astrocytoma and leptomeningeal disease.^22^ In our cohort, 7.5 % of the patients had leptomeningeal disease impacting on their overall survival, however the results were not statistically significant.

Individuals diagnosed with low-grade gliomas have comparatively better prognosis compared to their high-grade counterparts. In our cohort, patients with PA have a 1-year survival rate of 92 percent; additionally, the 5-year and 10-year survival rates were 87 and 83 percent, respectively. This was comparable to a study conducted by Abdalla et al at the Princess Norah Oncology Center, King Abdulaziz Medical City-Jeddah, where the 5-year overall survival was 90.6 percent.^23^

The mainstay of treatment in managing pilocytic astrocytoma is complete surgical resection, and the local recurrence rate is highly dependent on its success, as previously described in the literature. According to Fernandez et al., a total resection led to 100 percent 5- and 10-year survival, with a recurrence rate of 2-5.4 percent, while 42-45 percent of partially removed tumors showed recurrence.^11^ The 5 and 10-year survival in our study was 87 and 83 percent, respectively. The progression-free survival at 5 years was 10%, which was related to subtotal resection of the lesion, as most of the tumors were found in the infratentorial compartment, resulting in residual tumor, as complete resection would have increased the chances of neurological deficits. Additionally, some tumors in the supratentorial eloquent areas were not amenable to a GTR, adding to a lower progression-free survival rate.

In our cohort, 43 percent of the patients achieved gross total resection, while 8.1 percent of the patients achieved near-total resection, and 44.1 percent of the patients underwent subtotal resection. Biopsy was done only in limited patients accounting for 4.6 percent. The overall survival rates were higher in individuals who had gross total or near total resection. According to Johnson et al, among 865 patients with pilocytic astrocytoma, GTR was identified as a significant predictor of overall survival when compared to subtotal resection or biopsy.^10^ Additionally, Bond KM et al reported that individuals with a GTR were less likely to have a tumor recurrence than other surgical and non-surgical procedures.^24^

The role of postoperative radiation therapy has been controversial. It’s usually indicated in cases of incomplete resection, relapse of the tumor, or progression of an unresectable tumor and the patient’s age.^25^ Children more than 8 years of age can receive radiation if indicated along with chemotherapy if required. However, radiotherapy is contraindicated in individuals less than 8 years as it increases the risk of cognitive deficits along with endocrinological abnormalities and developmental defects.^26^ According to one study, multi-institutional database was reviewed from 1990-2016. Amongst these, a total of 141 patients were analyzed and 23 patients had a complete record about radiation induced toxicity.^27^ In our study, 84.3 percent of patients didn’t receive chemo or radiotherapy explaining its limited role in the management of these tumors.

The study has a retrospective design, which is a limitation, and therefore, further prospective studies will be helpful in explaining the overall prognosis for individuals suffering from Pilocytic Astrocytoma. Additionally, this is a single-center study, so multicenter studies would be beneficial.

## CONCLUSION

This institutional review of pilocytic astrocytoma provides insights into its clinical, radiological, and management aspects over the past decade in a low- and middle-income country with long-term follow-up. We reported overall and progression-free survival with 1-year, 5-year, and 10-year survival rates for the first time in our population. Survival trends show differences between various age groups, along with the impact of leptomeningeal disease, extent of resection, and radiological tumor patterns; however, the results were not statistically significant. On Cox regression, radiological patterns, postoperative CNS infection, endocrinological dysfunction, and visual symptoms were significantly predictive of OS. These trends reflect comprehensive, long-term follow-up with survival rates comparable to other centers. This study emphasizes that even in low- and middle-income countries, offering the best initial surgical resection is the most important prognostic factor in managing Pilocytic Astrocytoma cases. This understanding is especially important because the roles of radiation and chemotherapy are limited, particularly in resource-limited settings. Therefore, surgeons should aim to deliver their best efforts when working in such environments.

### Authors’ Contributions:

**SK:** Collected and analyzed the data, interpreted the results and drafted the manuscript.

**NA:** Interpreted the results and drafted the manuscript.

**FZ and UA:** Conducted literature search and data collection.

**AE:** Drafted the idea and supervised all the aspects of manuscript, reviewed it and final approval.
